# Erythropoietin as candidate for supportive treatment of severe COVID-19

**DOI:** 10.1186/s10020-020-00186-y

**Published:** 2020-06-16

**Authors:** Hannelore Ehrenreich, Karin Weissenborn, Martin Begemann, Markus Busch, Eduard Vieta, Kamilla W. Miskowiak

**Affiliations:** 1 Clinical Neuroscience, Max Planck Institute of Experimental Medicine, Göttingen, Germany; 2 Department of Neurology, Hannover Medical School, Hannover, Germany; 3 Department of Psychiatry & Psychotherapy, University Medical Center, Göttingen, Germany; 4 Center of Internal Medicine, Hannover Medical School, Hannover, Germany; 5 Institute of Neuroscience, University of Barcelona, IDIBAPS, CIBERSAM, Barcelona, Spain; 6 Psychiatric Centre Copenhagen, University Hospital, Rigshospitalet, Copenhagen, Denmark

**Keywords:** SARS-CoV-2; recombinant human erythropoietin, EPO, respiratory function, inflammation, cytokine storm, neuroprotection, clinical trial design

## Abstract

In light of the present therapeutic situation in COVID-19, any measure to improve course and outcome of seriously affected individuals is of utmost importance. We recap here evidence that supports the use of human recombinant erythropoietin (EPO) for ameliorating course and outcome of seriously ill COVID-19 patients. This brief expert review grounds on available subject-relevant literature searched until May 14, 2020, including Medline, Google Scholar, and preprint servers. We delineate in brief sections, each introduced by a summary of respective COVID-19 references, how EPO may target a number of the gravest sequelae of these patients. EPO is expected to: (1) improve respiration at several levels including lung, brainstem, spinal cord and respiratory muscles; (2) counteract overshooting inflammation caused by cytokine storm/ inflammasome; (3) act neuroprotective and neuroregenerative in brain and peripheral nervous system. Based on this accumulating experimental and clinical evidence, we finally provide the research design for a double-blind placebo-controlled randomized clinical trial including severely affected patients, which is planned to start shortly.

## Background

The COVID-19 *(coronavirus disease 2019, caused by severe acute respiratory syndrome coronavirus 2, SARS-CoV-2)* pandemic has reached yet unknown dimensions and is overwhelming societies, politics, medical systems and, in particular, intensive care units. The development of vaccines is ongoing but it still requires many months to years for them to be broadly accessible. Repurposed antiviral or immunomodulatory drugs and antisera are being evaluated in numerous, rapidly started clinical trials but thus far, despite perhaps some mild positive signals and even one speedy temporary approval by FDA and EMA (https://www.nih.gov/news-events/news-releases/nih-clinical-trial-shows-remdesivir-accelerates-recovery-advanced-covid-19;https://www.nature.com/articles/d41586-020-01295-8), no clearly effective candidate has hitherto evolved. Neutralizing monoclonal antibodies against proinflammatory cytokines and their respective receptors or complement inhibitors are considered as well, but all await clinical proof-of-concept studies (Bauchner and Fontanarosa 2020; Cao 2020; Del Rio and Malani 2020; Grein et al. 2020; Lythgoe and Middleton 2020; Mehta et al. 2020a; Sanders et al. 2020). First negative trials are already reported (Casadevall et al. 2020; Li et al. 2020a; Magagnoli et al. 2020).

A substantial number of affected individuals suffer a severe disease course, with some predominance in older individuals, but serious and fatal outcomes also increasingly observed in children and young adults. The grave cases show pneumonia with severe hypoxemia, requiring oxygen supply and mechanical ventilation (Li et al. 2020b; Liu et al. 2020; Mao et al. 2020; Nath 2020; Wu and McGoogan 2020; Yang et al. 2020) not infrequently combined with overshooting inflammatory reactions and a so-called ‘cytokine storm’ (Cao 2020; Allen et al. 2009; Gross et al. 2020; Liao et al. 2020; Mehta et al. 2020b; Wang et al. 2020a; Wen et al. 2020). More recently, an appreciable number of individuals with neurological complications has been identified, in particular among the severely affected subjects (Li et al. 2020b; Mao et al. 2020; Nath 2020; Avula et al. 2020; Desforges et al. 2019; Dube et al. 2018; Gandhi et al. 2020; Gu and Korteweg 2007; Helms et al. 2020; Moriguchi et al. 2020; Oxley et al. 2020; Toscano et al. 2020; Troyer et al. 2020). In the present therapeutic situation, which is essentially based on comprehensive general intensive care management, any additional measure to improve course and outcome of seriously afflicted individuals is of considerable importance. This review addresses the need and potential of symptom-targeting therapeutic measures.

## Introducing the candidate: recombinant human erythropoietin (EPO) – not only relevant for anemia treatment

Erythropoietin is a hypoxia-inducible growth factor, named after its original discovery in hematopoiesis (Jelkmann 1992; Krantz 1991). Over the last 30 years, it became more and more clear that EPO is expressed in many organs and tissues of the body, where it exerts multiple functions in the sense of a pleiotropic tissue-protective cytokine. EPO has not only successfully been used to treat or prevent anemia (the approved indication) but also for various other conditions, ranging from brain to different other organ diseases, in both human trials and numerous animal studies. Overall, in critically ill patients, EPO was safe and probably efficient, as summarized in recent meta-analyses (Litton et al. 2019; Mesgarpour et al. 2017).

Extension of EPO treatment to conditions other than anemia has not been appropriately supported by industry so far, partly due to expired patents, multiple biosimilar producers, fear of off-label use and of emerging additional side effects (Sargin et al. 2010). Therefore, its beneficial properties for treating e.g. brain disease could not be sufficiently demonstrated yet by large clinical trials needed for official approval of new indications. In the present COVID-19 pandemic, we suggest short-term supportive EPO treatment of severely affected patients, which we expect to improve disease course and outcome. Although case reports always call for extreme caution, two recently published/submitted case studies on EPO in seriously ill COVID-19 patients are encouraging for the present concept (Hadadi et al. 2020; Wincewicz et al. 2020, in review). In addition, potential supportive evidence is provided by the observation that hemodialysis patients with COVID-19 are likely to experience mild disease that does not develop into full-blown pneumonia. While the authors interpret this finding as possibly due to reduced function of the immune system and decreased cytokine storm in this patient group (Perico et al. 2020), we are rather inclined to see it related to their continuous EPO treatment.

## Three major levels of expected beneficial EPO action on respiratory function in severely affected COVID-19 patients

Severe acute respiratory syndrome of COVID-19 patients involves pulmonary and systemic inflammation, leading to multi-organ dysfunction in patients at high risk. Acute respiratory distress syndrome, sepsis, and acute cardiac decompensation are the most common critical complications during exacerbation. Approximately 15–33% of COVID-19 patients have severe course requiring intensive care, of whom up to > 30% need mechanical ventilation (Cao 2020; Del Rio and Malani 2020; Wu and McGoogan 2020; Goyal et al. 2020; Wang et al. 2020b). Chest radiographs are characterized by bilateral patchy infiltrates and chest computerized tomography scans demonstrate ground glass infiltrates. Histopathological findings in the lung include hyaline membrane formation, interstitial mononuclear inflammatory infiltrates, and multinucleated giant cells, findings similar to those in SARS or MERS coronavirus infections (Del Rio and Malani 2020; Munster et al. 2020). Brain invasion of SARS-CoV-2 may partially be responsible for the acute breathing failure of patients with COVID-19, with respiratory brainstem centers playing a prominent role. Associated neuropathy and myositis likely encompass phrenic nerve and respiratory muscles (Li et al. 2020b; Mao et al. 2020; Nath 2020; Lucchese and Floel 2020; Wu et al. 2020).

**Fig. 1:**
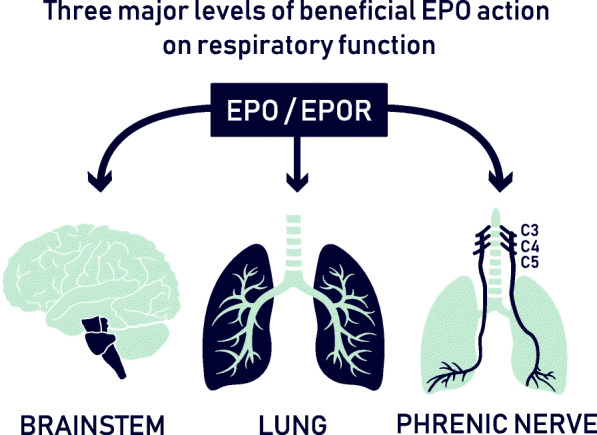
Beneficial pleiotropic EPO effects on respiratory function target several levels, comprising brainstem and central respiratory command, lung, including protection of overall tissue homeostasis, as well as phrenic motor facilitation and spinal plasticity in respiratory motor control

We propose that EPO will act on all three major levels of respiratory function in severely affected COVID-19 patients **(**Fig. 1)**.** Beneficial effects of EPO on the nervous system that could contribute to improved respiration include brainstem centers and phrenic nerve. For instance, EPO in the locus coeruleus attenuates the ventilatory response to CO_2_ in rats, i.e. it tunes the hypercapnia-induced hyperpnoea (Silva et al. 2017). EPO-mediated regulation of the central respiratory command involves MEK1/2 and PI3K (Caravagna and Soliz 2015). These pathways are also critical for phrenic motor facilitation induced by cervical spinal EPO, indicating that it elicits spinal plasticity in respiratory motor control under conditions of prolonged or recurrent low oxygen (Dale et al. 2012).

Several reports exist demonstrating beneficial EPO effects on acute lung injury and the acute respiratory distress syndrome (ARDS) in various different animal models (reviewed in (Kakavas et al. 2011)). EPO appears to exert its pleiotropic actions in the lung by protecting the integrity of the pulmonary epithelial and endothelial cells as well as by attenuating the associated pulmonary interstitial and alveolar epithelial edema and the deterioration of pulmonary oxygenation function (Kakavas et al. 2011; Zhu et al. 2019). This is achieved via modulating multiple levels of early signaling pathways involved in apoptosis, inflammation and peroxidation, potentially restoring overall homeostasis (Kakavas et al. 2011; Anagnostou et al. 1990; MacRedmond et al. 2009). Furthermore, EPO appears to confer vascular protection by promoting angiogenesis (Kakavas et al. 2011). Encouragingly, animal models of acute lung injury, consistently revealing beneficial EPO effects, comprise multiple etiologies. They include injury provoked by hyperoxia (Ozer et al. 2005), acute necrotizing pancreatitis (Tascilar et al. 2007), ischemia/reperfusion (Wu et al. 2009), sepsis induced by cecal ligation (Aoshiba et al. 2009) or by lipopolysaccharide (Shang et al. 2009), zymosan-induced non-septic shock (Cuzzocrea et al. 2006), tracheobronchial and pulmonary epithelial damage following brain trauma (Yildirim et al. 2005) and scald-burn inflammation (Rocha et al. 2015).

Data on EPO effects in human respiration are scarce. In a case of severe ARDS, lung function upon EPO improved and extracorporeal membrane oxygenation (ECMO) support could be reduced (Jungebluth et al. 2015). Intravenous EPO in volunteers exposed to mild hypoxia (10% O_2_ for 15 min) modulated the hypoxic ventilatory response. This effect appears to prevail via carotid body response, i.e. peripheral chemosensitivity, in humans as likewise seen before in mice (Soliz et al. 2009). Only marginally related, acute mountain sickness, characterized by headache, gastrointestinal symptoms, weakness, dizziness or lightheadedness, and difficulty sleeping in an unacclimatized person who recently arrived at an altitude above 2500 m, was prevented by prophylactic EPO injections (Heo et al. 2014). Efficacy of EPO versus placebo in critically ill patients, including those with lung disease admitted to intensive care, yielded reduction in allogeneic red blood cell transfusion. This trial did not evaluate effects on respiration, however, mortality and serious adverse events were not different between treatments, reassuring safety in pulmonary disease subjects (Silver et al. 2006).

## EPO to counteract inflammation and ‘cytokine storm’ in COVID-19

Pneumonia, lymphopenia, lymphocyte exhaustion markers and cytokine storm characterize severe COVID-19. CRP and D-dimer are abnormally high. Substantially elevated serum levels of proinflammatory cytokines, including IL-6, IL-1β, IL-2, IL-8, IL-17, G-CSF, GM-CSF and others, contribute to shock and multi-organ damage as well as to extremely diminished numbers of CD4+ T cells, CD8+ T cells, B cells, natural killer cells, monocytes, eosinophils and basophils (Cao 2020; Liao et al. 2020; Wen et al. 2020). In addition, SARS-CoV-2 infection of T cells could potentially induce T cell apoptosis (Wang et al. 2020a).

The nucleotide-binding domain and leucine-rich repeat-containing (NLR) family of pattern-recognition molecules mediate host immunity to various viral pathogens. Experiments utilizing analogs of dsRNA (poly-I:C) and ssRNA (ssRNA40) demonstrated that an NLRP3-mediated response could be activated by ‘virus mimetic’ RNA species (Allen et al. 2009). Whereas the NLRP3 inflammasome is an essential component in host defense against infection through sensing of viral RNA (Ivanov et al. 2020), its overshooting activity can be highly destructive. An excessive inflammatory response results in the progression of ARDS, with the NLRP3 inflammasome as key player.

Interestingly, EPO can effectively attenuate lung injury in mice by suppressing the NLRP3 inflammasome, which is dependent upon activation of EPOR/JAK2/STAT3 signaling and inhibition of the NF-kB pathway (Cao et al. 2020). This protective, balancing EPO mechanism might also be exploited for COVID-19 invasion into the brain, since in microglia, the NLRP3 inflammasome becomes (over) activated, too, when these cells sense virus. Subsequent caspase1 cleavage then markedly contributes to disease development and progression (Heneka et al. 2018).

EPO is probably in many instances of COVID-19 associated cytokine storm a better option than the ultima ratio immunosuppression (Mehta et al. 2020b), which may weaken, complicate or even endanger proper host defense. EPO suppresses proinflammatory cytokines, protects cells from apoptosis and promotes wound healing. EPO receptors (EPOR) are expressed on a variety of immune cells, enabling EPO to directly modulate their activation, differentiation and function (Peng et al. 2020; Suresh et al. 2019). Intriguingly, phagocyte respiratory burst activates macrophage EPO signaling to promote the resolution of acute inflammation (Luo et al. 2016). Both immunomodulation and anti-inflammation mediated by EPO promise another set of beneficial effects in severe COVID-19. In fact, combined use of EPO as anti-inflammatory as well as immunomodulatory treatment and antiviral drugs may even be more effective than using either one alone. Additionally, known side effects of antiviral drugs observed in SARS include anemia, which may improve upon EPO (e.g. (Fujii et al. 2004)).

## EPO: the case for neuroprotection in COVID-19

Neurologic manifestations of COVID-19 patients are increasingly reported, resemble symptoms/syndromes observed in SARS, occur most pronounced in severe cases, and range from headache, dizziness, impaired taste, smell or vision, ataxia, pain, nausea, delirium, seizures, meningoencephalitis, and impaired consciousness, to Guillain Barré syndrome, peripheral nervous system and skeletal muscle dysfunction/myositis (Mao et al. 2020; Nath 2020; Helms et al. 2020; Toscano et al. 2020; Wu et al. 2020; Baig 2020; Steardo et al. 2020). Very recently, a number of patients, many younger than 50 years, presented with radiographically confirmed acute stroke and PCR-confirmed SARS-CoV-2 infection, which emphasizes once more that neurological presentations of COVID-19 can be manifold (Avula et al. 2020; Oxley et al. 2020). Past viral pandemic-related outcomes also include neuropsychiatric symptoms, such as encephalopathy, mood changes, psychosis, or demyelinating processes, which accompanied acute viral infection or followed infection by weeks, months, or longer in recovered patients (Desforges et al. 2019; Moriguchi et al. 2020; Troyer et al. 2020; Moldofsky and Patcai 2011). Presence and persistence of human coronaviruses in human brain have been proposed to cause long-term sequelae (Troyer et al. 2020). All these observations are less surprising considering the viral spread via axons and neuron-to-neuron propagation, as known from SARS and other coronavirus infections in mammals (Dube et al. 2018; Gu and Korteweg 2007; Gu et al. 2005; Li et al. 2013; Netland et al. 2008). Coronaviruses may invade the CNS, disseminate, and participate in the induction of neurological diseases. Animal models revealed the route of neuropropagation from nasal cavity to olfactory bulb, piriform cortex and brainstem (Dube et al. 2018; Netland et al. 2008). Neurodestructive processes, including immune-mediated damage or exacerbated autoimmunity (Cao 2020; Toscano et al. 2020; Troyer et al. 2020; Lucchese and Floel 2020; Moldofsky and Patcai 2011; Lo et al. 2006; Zhang et al. 2020) may thereby well be initiated and contribute to neurodegenerative diseases like Morbus Alzheimer and other dementias as late consequences. Therefore, neuroprotective strategies should be initiated in severely affected COVID-19 patients without any delay.

EPO appears as a well-suited candidate to provide the required lasting and comprehensive neuroprotection. In the mammalian brain, EPO and EPO receptor (EPOR) expression is upregulated upon pathological conditions, e.g. brain injury of different etiologies, where it exerts anti-apoptotic, neuroprotective and neuroregenerative effects, independent of hematopoiesis (Brines and Cerami 2005; Digicaylioglu et al. 1995; Marti et al. 1996; Shingo et al. 2001). Taking an unusual reverse approach (human trials first), we reported that EPO treatment has potent neuroprotective and procognitive properties in patient groups as different as ischemic stroke, chronic schizophrenia, chronic progressive multiple sclerosis, treatment-resistant major depression and bipolar disease (Ehrenreich et al. 2007a; Ehrenreich et al. 2007b; Miskowiak et al. 2014a; Miskowiak et al. 2014b). In schizophrenia and affective disorders, we even detected in independent trials reduction of grey matter loss upon EPO (Miskowiak et al. 2015; Wustenberg et al. 2011). Animal studies performed over more than two decades confirmed these beneficial effects in a multitude of different disease models and started to provide mechanistic insight into the (patho) physiological role of the endogenous brain EPO system which includes strong effects on neurodifferentiation and neuroplasticity (Sargin et al. 2010; Sakanaka et al. 1998; Wakhloo et al. 2020). To achieve sufficiently high neuroprotective concentrations in the brain - independent of an intact or during COVID-19 potentially compromised blood-brain-barrier - high-dose intravenous EPO must be recommended as successfully used in our clinical studies on brain diseases mentioned above. To avert possible hematopoietic side effects of EPO, routine laboratory screening and - if indicated - preventive measures accompany each EPO application (for detailed description see (Bartels et al. 2008)). Taken together, EPO might have the potential to improve outcome of COVID-19 patients regarding acute as well as chronic-progressive downstream sequelae of the central and peripheral nervous system.

## EPO indications outside the hematopoietic system: difficult ever since - but worthwhile pursuing

After > 20 years of own experience in translational work on the brain EPO system, including many clinical trials, we feel that the following points need to be addressed in the context of this review and before presenting our design of a proof-of-concept trial on EPO treatment in COVID-19 below.

Work on EPO indications outside the hematopoietic system has been challenging ever since, not only because of difficulties obtaining funding as above mentioned briefly. We had to learn the downstream consequences of pharmaceutical companies and subsequently regulatory bodies drawing premature conclusions based on too superficially or not at all analyzed data. This did not only damage further work on EPO in stroke (Ehrenreich et al. 2009), but also influenced other EPO trials negatively (e.g. (Grasso et al. 2016)). To add to the problematic situation, studies with suboptimal trial/endpoint design despite preexisting knowledge clearly did not benefit the ‘overall reputation of EPO’ (Ehrenreich et al. 2020). Scientifically unfounded conclusions triggered > 10 years ago an avalanche of destruction regarding the German EPO stroke multicenter trial where inclusion/treatment violations of stroke patients in most of the recruiting centers (totally independent of the study medication) explained the outcome rather than EPO itself (Ehrenreich et al. 2009). In fact, careful subpopulation analysis of all deceased patients revealed that several relevant baseline characteristics (i.e. data obtained before administration of any study medication) were significantly different between groups, always in disadvantage of the EPO group. For instance, upon inclusion (before study drug application), intent-to-treat non-rtPA patients receiving EPO, who died, suffered from much severer strokes as compared to placebo patients (NIHSS day 1: 20.4 ± 5.4 versus 13.3 ± 4.9; *p* = 0.003). This highly significant prediction of a worse outcome explains the twofold higher very early death rate in the EPO group (for more information, visit http://www.epo-study.de/index_eng.html).

In addition, the multicenter EPO stroke trial had to run over several years due to lack of funding in between, and exactly during this time, rtPA treatment in Germany rose dramatically (including numerous violations of rtPA indications in all but one of the trial centers). This certainly added much to the overall negative trial outcome. Importantly, we note that regarding non-rtPA patients, the originally reported benefit of EPO (Ehrenreich et al. 2002) was fully reproduced in this multicenter trial (Ehrenreich et al. 2009). In fact, not only the outcome of this non-rtPA subgroup (Ehrenreich et al. 2009) and the first EPO stroke trial (Ehrenreich et al. 2002) were promising. Also the retrospective analysis of patients only from Hannover, the most efficiently recruiting center of the multicenter EPO stroke trial, with essentially no violations of inclusion criteria and *lege artis* rtPA treatment, made the beneficial effect of EPO in stroke (independent of rtPA) once more obvious (Worthmann et al. 2013).

Longer treatment duration - over many weeks - may ultimately enhance the benefit of EPO for neuroprotection and neuroregeneration also after stroke. Clinical studies on EPO in chronic brain diseases (schizophrenia, multiple sclerosis, major depression and bipolar disorder) with extended treatment using high-dose EPO over many weeks showed consistently advantageous effects on cognition, motor function, and even reduction of brain matter loss (Ehrenreich et al. 2007a; Ehrenreich et al. 2007b; Miskowiak et al. 2014a; Miskowiak et al. 2014b; Miskowiak et al. 2015; Wustenberg et al. 2011). All these findings were in absence of any noticeable side effects. Therefore, in light of more and more reports on and predictions of long-term consequences of COVID-19 in the sense of a virus-induced post-infection neurodegenerative course (delineated in the previous section), not only the potential for acute treatment of SARS-CoV-2 by EPO, but also the substantial chance for chronic treatment might be worthwhile considering.

Of course, in all clinical EPO studies, the quality of patient care including alert follow-up of individual patients at all times is mandatory (Bartels et al. 2008; Siren et al. 2009). EPO is a potent growth factor, not a miracle drug, and it is no causal treatment or cure of brain diseases but it may improve their outcome.

## Design of a proof-of-concept trial on EPO treatment in COVID-19

**Fig. 2:**
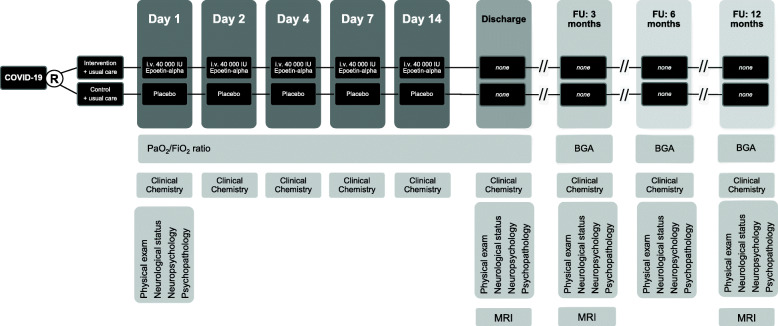
Design of a double-blind, placebo-controlled, randomized proof-of-concept trial (phase IIb) on EPO treatment in severely affected COVID-19 patients. Patients receive 40,000 IU recombinant human erythropoietin (EPO) or placebo plus standard care on the first day of mechanical ventilation (day 1), as well as on days 2, 4, 7 and 14. Pulmonary function and laboratory work-up including inflammatory parameters are monitored throughout in-hospital treatment, as well as on follow-up examinations at 3, 6 and 12 months after discharge. At discharge and after 3 and 12 months, magnetic resonance imaging (MRI) and spectroscopy (MRS) of the brain take place. BGA = blood gas analysis, FU = follow-up

Based on the experimental and clinical studies on EPO summarized in the above sections, we hypothesize that EPO treatment has positive effects on clinical course and outcome of critically ill COVID-19 patients. A proof-of-concept study of EPO in COVID-19 is therefore in preparation (Fig. 2)**.**

In this planned double-blind, placebo-controlled, randomized proof-of-concept (phase IIb) trial with safety/futility assessment following enrollment of 20 and 40 patients, respectively, the proportion of patients with clinical improvement from WHO R&D Blueprint ordinal scale grade 6 (hospitalized, on invasive mechanical ventilation or ECMO) to grade 5 (hospitalized, on non-invasive ventilation or high flow oxygen devices) on day 14 will be the primary outcome. EPO treatment will start as soon as the critical state requiring mechanical ventilation is reached. Secondary outcomes include COVID-19 mortality, all-cause mortality, time (days) to improve in clinical status from critical (WHO grade 6) to stable (WHO grade 5), length of hospital stay for those who recover, conversion rate of clinical status on day 7 after treatment start, duration of ECMO, duration of vasopressors, duration of oxygen therapy, SOFA score, main blood cell counts (including lymphocyte subsets via FACS), levels of CRP, LDH, D-dimer, ferritin, Il-6 and other cytokines at days 4, 7 and 14 after start of treatment, as well as delta CRP, LDH, D-dimer, ferritin, Il-6 and other cytokines at days 4, 7 and 14 compared to baseline at start, PaO2/FiO2 ratio on days 1, 2, 3, 4, 7 and 14 after treatment start. Blood for analyses will always be drawn before EPO/placebo injection.

Inclusion criteria are SARS-CoV-2 infection confirmed by PCR, age > 18 years, all genders, respiratory failure (PaO2/FiO2 < 300 or SatO2/FiO2 < 220) demanding mechanical ventilation. Exclusion criteria are evidence of terminal chronic end organ failure (renal, cardiac, hepatic, gastrointestinal), thrombocytosis, life expectancy ≤24 h, and concomitant potentially serious infections.

The intervention consists of intravenous application of recombinant human EPO (40,000 IU) or placebo at start of mechanical ventilation (day 1), repeated 24 h (day 2) and 72 h (day 4) later, as well as on days 7 and 14 (cumulative dose of 200,000 IU per patient). In addition, all patients receive standard intensive care anticoagulation (heparin), as anyhow strongly advisable for COVID-19 patient care (Levi et al. 2020; Paranjpe et al. 2020), and can otherwise obtain any treatment considered necessary for their clinical management.

Patients shall have follow-up examinations during their hospital stay, daily while on mechanical ventilation, and twice weekly thereafter (clinical and laboratory parameters) until discharge from hospital. Further clinical follow-up shall be performed at 3, 6 and 12 months after discharge, including assessment of pulmonary function and inflammation markers, neurological status, neuropsychological (cognitive) and psychopathological assessment, if possible combined with MRI/MRS of the brain at discharge as well as 3 and 12 months thereafter.

## Conclusion

In this brief review, we address COVID-19 and the need of symptom-targeting therapeutic measures in the present pandemic situation where convincingly efficient antiviral drugs and vaccination are still absent. We present in brief chapters the major problems of severely affected COVID-19 patients and delineate the potential of EPO to relieve them. We conclude this article with a clinical research design as basis of a planned clinical trial, which is supposed to start shortly.

## Data Availability

Not applicable.

## Abbreviations

ARDS: Acute respiratory distress syndrome
COVID-19: Coronavirus disease 2019
CRP: C-reactive protein
dsRNA: Double-stranded RNA
ECMO: Extracorporeal membrane oxygenation
EMA: European Medicines Agency
EPO: Erythropoietin
EPOR: Erythropoietin receptor
FACS: Fluorescent antibody cell sorting
FDA: Food and Drug Administration
G-CSF: Granulocyte-colony stimulating factor
GM-CSF: Granulocyte-macrophage colony-stimulating factor
IL: Interleukin
LDH: Lactate dehydrogenase
MERS: Middle East respiratory syndrome-related coronavirus
MRI: Magnetic resonance imaging
MRS: Magnetic resonance spectroscopy
NLR: Nucleotide-binding domain and leucine-rich repeat-containing family
PaO2/FiO2: Ratio of arterial oxygen partial pressure (PaO2 mmHg) to fractional inspired oxygen (FiO2)
R&D: Research and development
rtPA: Recombinant tissue plasminogen activator
SARS: Severe acute respiratory syndrome
SARS-CoV-2: Severe acute respiratory syndrome caused by coronavirus 2
SatO2/FiO2: Ratio of oxyhemoglobin saturation in arterial blood to fractional inspired oxygen (FiO2)
SOFA: Sepsis-related organ failure assessment score
ssRNA: Single-stranded RNA
WHO: World Health Organization
WHO R&D Blueprint: Global strategy and preparedness plan that allows the rapid activation of R&D activities during epidemics. Its aim is to fast track the availability of effective tests, vaccines and medicines that can be used to save lives and avert large-scale crisis. Classification of disease severity based on WHO R&D Blueprint (WHO grades)
